# Identification of Calcium Channel-Related Gene P2RX2 for Prognosis and Immune Infiltration in Prostate Cancer

**DOI:** 10.1155/2022/8058160

**Published:** 2022-09-30

**Authors:** Qinyu Li, Bili Wu, Motuma Daba, Xintao Gao, Bingliang Chen, Guoda Song, Kai Zeng, Jianping Miao, Xianglin Yuan, Jihong Liu, Zhihua Wang, Bo Liu

**Affiliations:** ^1^Department of Urology, Tongji Hospital, Tongji Medical College, Huazhong University of Science and Technology, Wuhan, 430030 Hubei, China; ^2^Department of Oncology, Tongji Hospital, Tongji Medical College, Huazhong University of Science and Technology, Wuhan, 430030 Hubei, China; ^3^Department of Geriatrics, Tongji Hospital, Tongji Medical College, Huazhong University of Science and Technology, Wuhan, 430030 Hubei, China

## Abstract

Prostate cancer is one of the most common malignancies in men. Calcium signaling is implicated in the progression of prostate cancer and plays a critical role in immune cell function. However, whether specific calcium channel-related genes play a crucial role in the immune cell infiltration levels of prostate cancer requires further research. In this study, we performed an integrated analysis of transcriptional, clinical, and somatic mutation data from The Cancer Genome Atlas database and identified the hub calcium channel-related gene P2RX2 to be associated with the prognosis and immune infiltration of prostate cancer. P2RX2 expression was positively correlated with immune cell infiltration levels and the expression of immune checkpoint genes, and downregulation of P2RX2 led to poor survival in patients with prostate cancer. Furthermore, we validated the molecular and clinical characteristics of P2RX2 by using multiple databases and conducting in-vitro experiments. Additionally, drug sensitivity analysis revealed that patients with low P2RX2 expression were sensitive to docetaxel and Bicalutamide. In conclusion, we revealed an association between calcium channel-related genes and prostate cancer, and identified P2RX2 as a biomarker for early diagnosis, prognosis prediction, and aiding treatment decisions for patients with prostate cancer.

## 1. Introduction

Prostate cancer (PCa) is the most frequently diagnosed cancer in men [1]. Metastasis accounts for most cancer-related deaths and is difficult to manage [2, 3]. The five-year survival rate of metastatic PCa is significantly lower than that of local PCa [4]. Metastatic dissemination reportedly occurs in the early stages of cancer, though clinical manifestations often take years [5]. However, the mechanisms underlying the pathogenesis and metastasis of PCa remain poorly understood. Therefore, a better understanding of molecular dysfunction in cancer and the identification of effective biomarkers that can predict the prognosis of patients and serve as targets for the treatment of PCa are crucial.

Calcium signaling has been reported to be instrumental in the development of PCa and involved in tumor progression at different stages [6, 7]. Several calcium channels contribute to the promotion of PCa cell survival. Enhanced Orai3 protein expression is associated with PCa progression, and the Orai3–Orai1 channel predominance contributes to apoptotic resistance and enhanced proliferation of PCa cells [8]. For some specific hub genes, the upregulation of TRPM2 is correlated with alterations in autophagy, leading to an improvement in the survival of PCa cells [9]. Moreover, some characteristics of tumor cells, such as migration and invasion, can be regulated by TRPM4 expression [10]. Another calcium channel-related gene, TRPV6, is overexpressed in multiple types of human malignancies, and its upregulation is strongly correlated with tumor progression and metastasis, resulting in poor survival of patients [11].

Currently, common treatments for PCa include surgery, androgen deprivation therapy, radiation therapy, chemotherapy, and immunotherapies [12]. Immunotherapy has revolutionized cancer treatment in recent years. To date, patient responsiveness to immunotherapy has failed to yield satisfactory results in patients with PCa. Although a small proportion of patients have been observed to have durable clinical responses, the majority of them are unable to benefit from immunotherapy. The tumor microenvironment reportedly affects the clinical outcomes of immunotherapy. Interestingly, several studies have reported that calcium, which acts as a second messenger to regulate intracellular signaling pathways [13], plays a fundamental role in immune cell function [14] and has an impact on the proliferation, differentiation, apoptosis, and transcription of numerous genes [15]. Nevertheless, whether specific calcium channel-related genes play a significant role in the immune cell infiltration levels of PCa is yet to be elucidated.

In this study, we performed a comprehensive analysis of public data from The Cancer Genome Atlas (TCGA) database and identified the hub calcium channel-related gene P2RX2, which was associated with the prognosis and immune cell infiltration of PCa. We validated the role of P2RX2 by using multiple databases and conducting molecular experiments. Moreover, we performed pan-cancer and drug sensitivity analyses of P2RX2 for better clinical applications.

## 2. Materials and Methods

### 2.1. Data Collection and Differential Analysis

This study was approved by the Institutional Research Ethics Committee of Tongji Hospital. Transcriptional, clinical, and somatic mutation data were obtained from TCGA database. The calcium channel-related genes were obtained from the Gene Ontology (GO) website using the keyword “calcium channel.” Transcriptome data were processed using the “edgeR” package [16]. Differentially expressed genes (DEGs) with |log2FC| >1, and *p* values <0.05 were selected for further analysis.

### 2.2. GO and KEGG Enrichment Analyses

To further delve into the biological functions of the differentially expressed calcium channel-related genes, GO analysis, including molecular function, cellular component, and biological process, and Kyoto Encyclopedia of Genes and Genomes (KEGG) pathway enrichment analysis were conducted using the R package “clusterProfiler” [17] with thresholds of adjusted *p* value <0.05 and *q* value <0.05.

### 2.3. Identification of Hub Genes

To recognize the prognostic value of DEGs, univariate Cox regression and LASSO regression analyses were performed to obtain candidate genes with a *p* value <0.05 between progression-free survival (PFS) and gene expression levels. Then, the selected genes were put into the step multivariate Cox regression model. Thereafter, we applied “ssGSEA” to estimate the immune cell infiltration levels by using the R package “GSVA” [18]. The correlation between immune infiltration degrees of immune cells, expression of immune checkpoint genes, androgen receptor, and the prognostic genes were analyzed.

### 2.4. Relationship between P2RX2 Expression and Clinical Parameters

To further investigate the potential role of P2RX2, we used the GEPIA database [19] to confirm the differential expression between tumor and normal tissues by matching GTEx data and conducted survival analysis of P2RX2. The associations between P2RX2 expression and various clinicopathological parameters (TNM stages, Gleason score, and progression-free interval [PFI] events) of PCa were assessed. Finally, cBioPortal [20] was utilized to reveal the relationship between biochemical recurrence-free survival time and P2RX2 expression.

### 2.5. Genetic Alterations of P2RX2

To explore gene mutations of P2RX2 in PCa, the cBioPortal was used to evaluate the alteration frequency of P2RX2. The COSMIC database [21] was also used to identify the types of gene mutations. Finally, somatic mutation data downloaded from TCGA database were analyzed to visualize the mutation landscape between the P2RX2^high^ and P2RX2^low^ groups by using the “Maftools” package [22].

### 2.6. Coexpression Analysis of P2RX2

Coexpression analysis was performed using LinkedOmics [23], which contains multiomics data for 32 cancer types, and the results are demonstrated in volcano plots and heatmaps. Moreover, the GO annotation and KEGG pathways of the coexpressed genes of P2RX2 were performed using the “LinkInterpreter” module.

### 2.7. Immune Infiltration and Tumor Mutation Burden Analysis

To perform immune infiltration analysis, we used the estimate algorithm in the R package “estimate” [24] to calculate the ImmuneScore and StromalScore. Next, the association between the expression of P2RX2 and tumor-infiltrating immune cells was assessed using the TIMER database [25]. We also estimated the correlation between immune checkpoint gene expression and P2RX2 expression. In calculating the immunophenoscore (IPS), four categories of immunogenicity-determining genes (effector cells, immuno-suppressor cells, MHC molecules, and immune modulators) were evaluated [26]. Therefore, we compared the IPS of patients in the P2RX2^high^ and P2RX2^low^ groups to infer the potential response of patients to immunotherapy. Since tumor mutation burden (TMB) has been considered a biomarker of prognosis and response to immunotherapy in cancer [27], we evaluated the TMB between the P2RX2^high^ and P2RX2^low^ groups. Finally, the expression of P2RX2 in different immune subtypes was explored using the TISIDB database [28].

### 2.8. Drug Sensitivity Analysis

The NCI-60 human tumor cell line panel contains drug sensitivity and molecular and phenotypic data for various cancers. CellMiner is a web application that provides tools to acquire NCI-60 data [29, 30]. RNA-seq and DTP NCI-60 data were downloaded, and drugs with FDA approval or those undergoing clinical trials were selected for further analysis. The correlation between P2RX2 expression and drug sensitivity was estimated. Additionally, we downloaded the transcriptional data of tumor cell lines and IC50 values of antitumor drugs from the Genomics of Drug Sensitivity in Cancer (GDSC) database [31] and performed drug sensitivity analysis with the “pRRophetic” package [32].

### 2.9. Pan-Cancer Analysis of P2RX2

To explore the possible roles of P2RX2 in other cancers, we conducted a pan-cancer analysis of P2RX2. TIMER was used to demonstrate differential expression in the 33 cancer types. Furthermore, RNA-seq data of the 33 cancer types in the TCGA database were downloaded to evaluate the correlation between P2RX2 and immune cells. Associations with immune checkpoints, such as SIGLEC15, IDO1, PD-L1, HAVCR2, PD1, CTLA4, LAG3, and PD-L2, were also assessed.

### 2.10. Gene Set Enrichment Analysis

GSEA was performed to determine the differences in signaling pathways between the P2RX2^high^ and P2RX2^low^ groups to reveal the biological functions of P2RX2. Nominal *p* values <0.05 and FDR *q* values <0.25 were considered statistically significant.

### 2.11. Immunohistochemical Staining

Immunohistochemical staining was performed on paraffin sections prepared from the clinical specimens at our hospital. Rabbit anti-P2RX2 (DF13236; Affinity Biosciences) and goat antirabbit secondary antibodies (GB23303; Servicebio) were used. A DAB kit was used for visualization after incubation with the secondary antibody. Subsequently, the sections were counterstained with hematoxylin and analyzed using a bright-field microscope equipped with a digital camera (Nikon, Japan).

### 2.12. Validation of P2RX2 in Molecular Experiments

Two PCa cell lines (CWR22Rv1 and C4-2b) were purchased from the Shanghai Institute of Cell Biology (Shanghai, China) and Wuhan Shanen Biotechnology (Wuhan, China). The cell lines were cultured in RPMI1640 medium (Wuhan Boster Biological Technology, China) with 10% fetal bovine serum (Gibco, Invitrogen, Shanghai, China) and incubated at 37°C in a 5% CO_2_ incubator at a constant temperature. C4-2b and CWR22Rv1 cells were seeded in 6-well plates for transfection. P2RX2-overexpressing plasmids and corresponding control vectors were generated by Wuhan Viral therapy Technologies (Wuhan, China), and the effective sequences are presented in Table S1. P2RX2-overexpressing plasmids and no-load control plasmids were transfected using Lipofectamine 3000 (Invitrogen, Shanghai, China). Thereafter, protein samples were extracted from the transfected cells, subjected to SDS-PAGE, separated by electrophoresis, and transferred onto PVDF membranes. The membranes were blocked in 5% BSA and subsequently incubated with primary antibodies against P2RX2 (DF13236, Affinity) and GAPDH (A00227-1, Wuhan Boster Biological Technology) at 4°C overnight. The membranes were then washed thrice with TBST three times and incubated with secondary antibodies at room temperature for 1 h.

To validate the tumor-suppressive role of P2RX2 in PCa, cell activity was detected using a cell counting kit-8 (CCK-8) assay (C0037, Beyotime). The cells were seeded into 96-well plates, and a CCK-8 solution was added to each well after 24 h. The absorbance of each well at 450 nm was measured after 2.5 h. Next, a colony formation assay was performed. The cells were seeded in 6-well plates (2 × 104/well) and cultured for 2 weeks. The cells were fixed in formaldehyde and stained with crystal violet, and cell clones were counted. Finally, a scratch assay was performed. The cells were seeded in 24-well plates and incubated until 100% confluence was reached. After 72 h, the changes in cell migration were estimated.

### 2.13. Statistical Analysis

All analyses were performed using RStudio 4.0.4. Correlation analysis was performed using Spearman method. Student's *t*-test and Wilcoxon test were used for two-group comparisons. Correspondingly, the Kruskal–Wallis test was used for multiple groups. Statistical significance was set at *p* < 0.05.

## 3. Results

### 3.1. Differential Expression, GO, and KEGG Analyses

**Figure 1:**
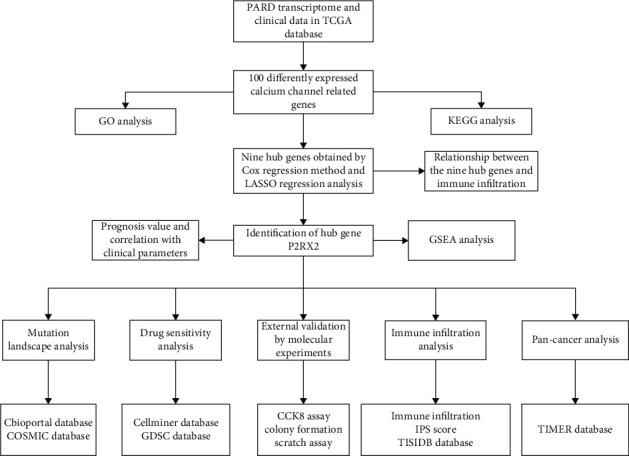
Flow diagram of this study.

**Figure 2:**
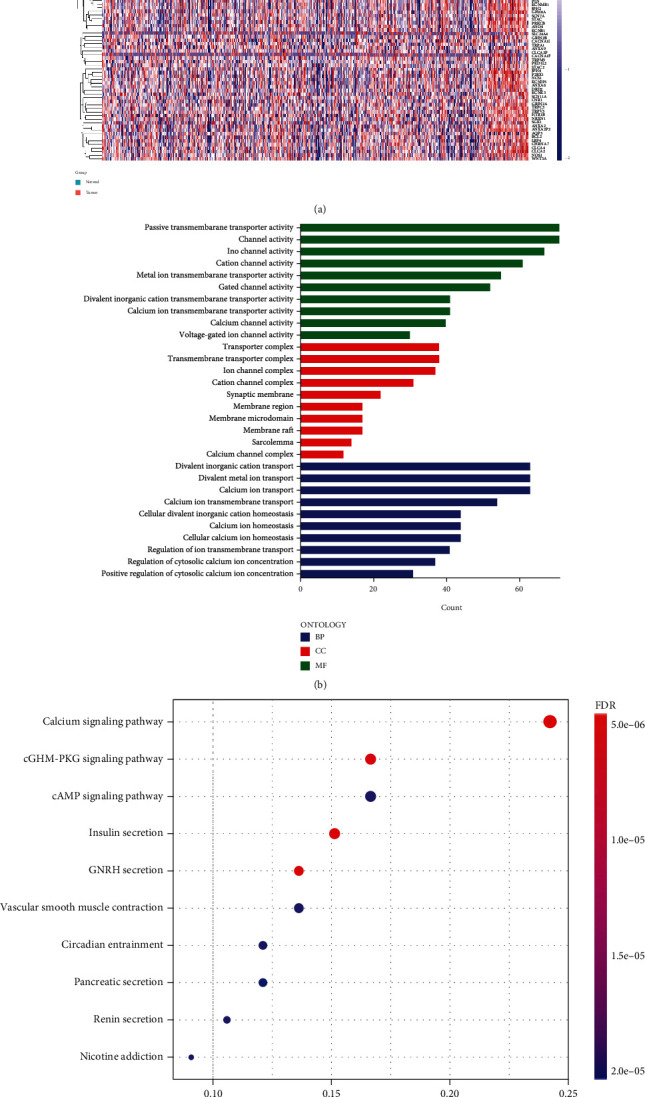
The differentially expressed calcium channel-related genes and the functional enrichment analysis: (a) A heat map of the differentially expressed calcium channel-related genes between prostate cancer and normal tissues. (b) GO analysis of differentially expressed genes (BP: biological processes; CC: cellular components; MF: molecular function). (c) KEGG analysis of differentially expressed genes.

The flow diagram of the study is presented in Figure 1. In total, 100 significantly differentially expressed genes, including 77 downregulated and 23 upregulated genes, were identified by differential analysis. The heat map (Figure 2(a)) and volcano plot (Figure S1) demonstrate the expression distribution of the dysregulated calcium channel-related genes. GO and KEGG enrichment analyses were performed to reveal the biological roles of dysregulated calcium channel-related genes (Figures 2(b) and 2(c)). Calcium channel-related genes are mainly involved in biological processes, including divalent inorganic cation transport, divalent metal ion transport, and calcium ion transport. Cellular components are mainly associated with the transporter complex and transmembrane transporter complex. Moreover, molecular functions are significantly enriched in passive transmembrane transporter and channel activities. The KEGG pathway analysis revealed that calcium channel-related genes mainly participate in the calcium, cGMP-PKG, and cAMP signaling pathways.

### 3.2. Identification of the Hub Gene

**Figure 3:**
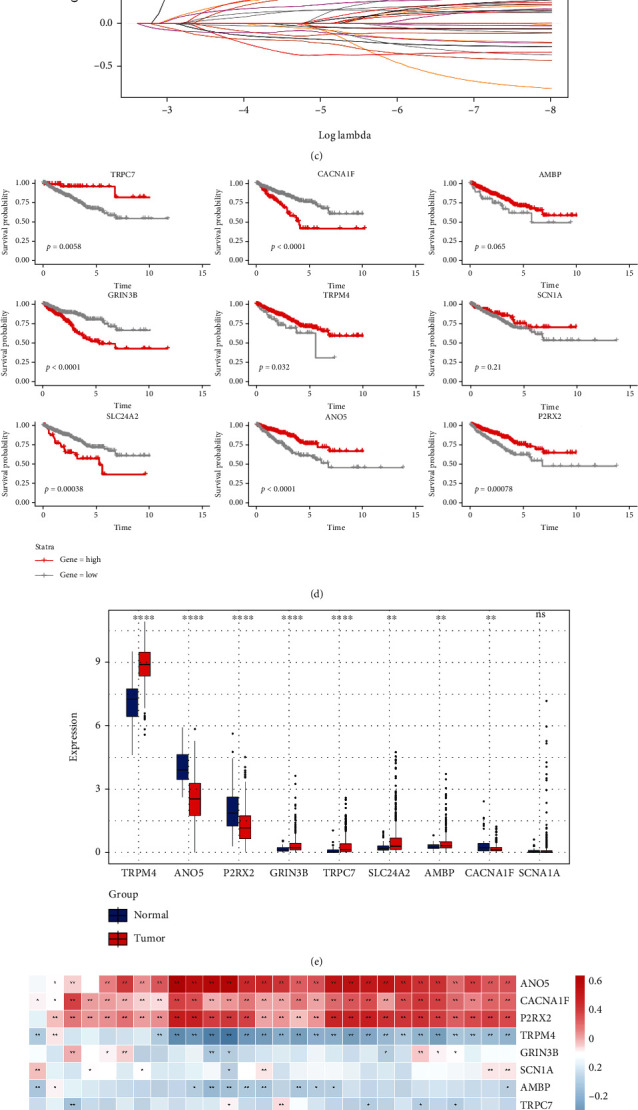
Identification of the P2RX2 gene: (a) Univariate Cox regression analysis of differentially expressed genes. (b–c) LASSO regression analysis of the genes correlated with progression-free survival (PFS) in univariate Cox regression analysis. (d–f) Kaplan–Meier PFS curve analysis (d) and the expression between tumor and normal tissues (e) of the nine hub genes. (f–g) The correlation of immune cell infiltration levels (f), immune checkpoint genes (g), and the nine hub genes. (h) The association between the expression of immune checkpoint genes and androgen receptors and P2RX2 expression (^∗^*p* < 0.05, ^∗∗^*p* < 0.01, ^∗∗∗^*p* < 0.001, ^∗∗∗∗^*p* < 0.0001).

After univariate Cox regression analysis, 29 differentially expressed genes remained (Figure 3(a)). A Lasso regression analysis was performed to further filter the 14 prognostic genes (Figures 3(b) and 3(c)). Nine prognostic genes (TRPC7, CACNA1F, AMBP, GRIN3B, TRPM4, SCN1A, SLC24A2, ANO5, and P2RX2) remained after the multivariate Cox regression analysis. Survival and differential analyses between tumor and normal tissues performed well in terms of prognostic genes (Figures 3(d) and 3(e)). Since calcium signals play a crucial role in immune cell functions, we correlated prognostic genes with infiltrating immune cells. The results revealed that ANO5, CACNA1F, and P2RX2 were closely correlated with infiltrating immune cells (Figure 3(f)). As both checkpoint genes and AR play a critical role in PCa progression, the correlation between them and prognostic genes was evaluated (Figure 3(g)). Of these, ANO5, CACNA1F, and P2RX2 (Figure 3(h)) were particularly significant, suggesting their potential role in predicting clinical response to immunotherapy. However, the expression of CACNA1F is very low in both tumor and normal tissues, and the prognostic value of ANO5 in PCa has previously been investigated [33]. Hence, we selected P2RX2 for further analysis.

### 3.3. Relationship between P2RX2 Expression and Clinical Parameters

**Figure 4:**
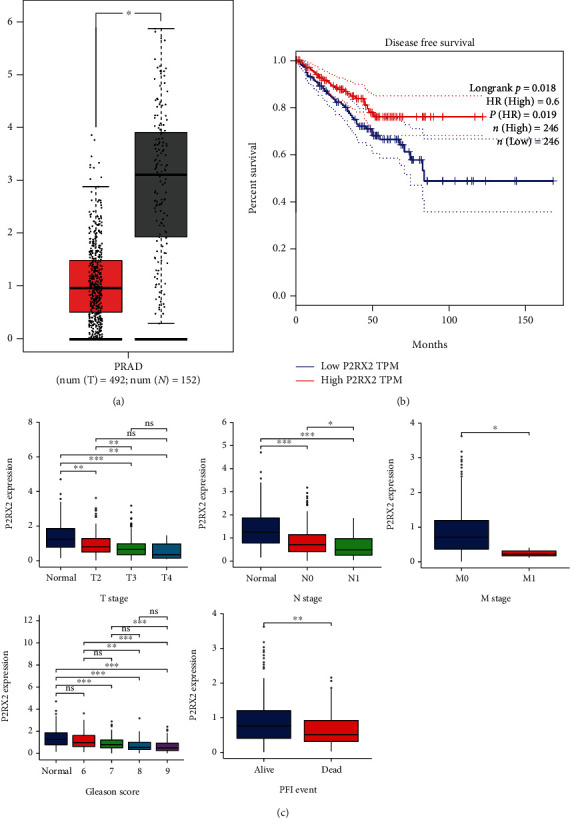
Clinical significance of P2RX2: (a) The differential expression of P2RX2 in PCa compared with normal samples in TCGA and GTEx datasets. (b) Association between the expression of P2RX2 and DFS in GEPIA. (c) Correlations of P2RX2 expression and *T* stage, *N* stage, *M* stage, Gleason score, and PFI events (^∗^*p* < 0.05, ^∗∗^*p* < 0.01, ^∗∗∗^*p* < 0.001).

To uncover the clinical relevance of P2RX2, we first used GEPIA to compare its expression between tumor and normal tissues, and the results matching GTEx demonstrated that the expression of P2RX2 was very low in PCa (Figure 4(a)). Furthermore, the survival curves demonstrated that lower P2RX2 expression was significantly associated with lower PFS (HR = 0.6, *p* = 0.018) (Figure 4(b)). Additionally, the expression of P2RX2 with different biochemical recurrence-free (BCR) statuses were compared (Figure S2), which also indicated that P2RX2 may be a protective factor for patients with PCa. Moreover, P2RX2 expression was verified by clinical parameters, and the results indicated that decreased P2RX2 expression levels were remarkably correlated with TNM stages, Gleason score, and PFI events (Figure 4(c)). The correlations between P2RX2 expression and the clinicopathological parameters are presented in Table 1. As depicted in Figure S3, P2RX2 expression was significantly decreased in PCa with a high Gleason score.

### 3.4. Gene Mutations of P2RX2 in PRAD

**Figure 5:**
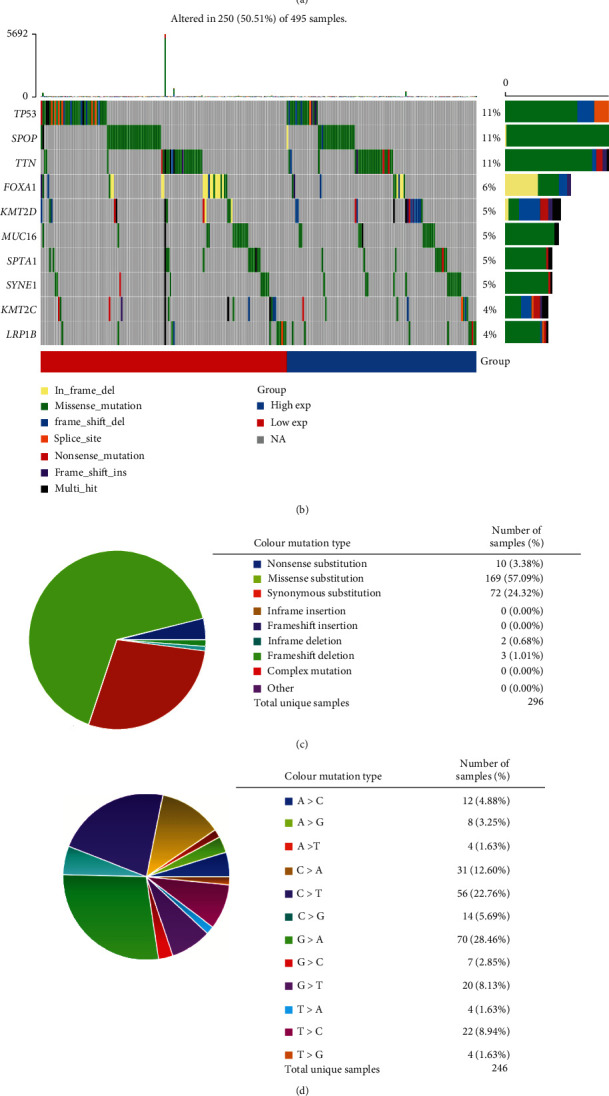
Gene mutations of P2RX2 in PCa: (a) OncoPrint of P2RX2 mutations in the TCGA-PRAD cohort (cBioPortal). (b) Waterfall plot displaying the mutation status of genes with high mutation frequencies in the P2RX2^high^ and P2RX2^low^ groups. (c–d) The mutation types of P2RX2 in PCa.

To better understand the biological functions of P2RX2, coexpression analysis was conducted using LinkedOmics, and 6205 genes were positively correlated, while 3876 genes were negatively correlated with P2RX2 (Figure 5(a)). The top 50 positively and negatively correlated genes are presented in Figures 5(b) and 5(c), respectively. The results of the enrichment analysis indicated that P2RX2 coexpressed genes were mainly involved in biological processes, including the regulation of chemotaxis. The KEGG pathway analysis revealed that P2RX2 coexpressed genes mainly participated in aminoacyl-tRNA biosynthesis, homologous recombination, and DNA replication (Figure 5(d)).

### 3.5. Coexpression Analysis of P2RX2

**Figure 6:**
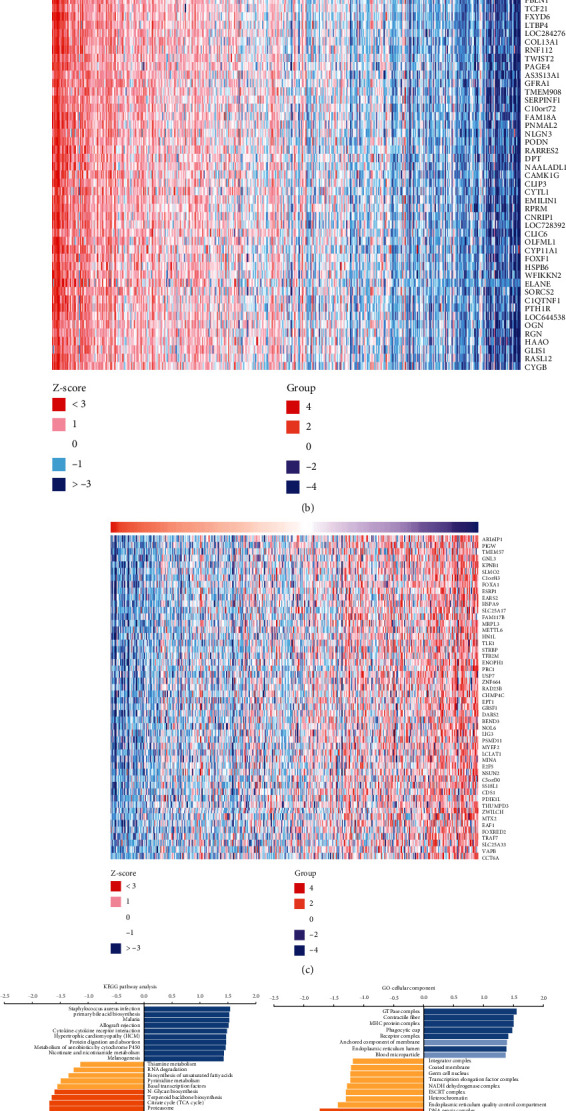
The coexpression networks of P2RX2 in PCa. (a) Volcano plot of the genes significantly correlated with P2RX2. (b–c) Heatmap of the top 50 genes positively (b) and negatively (c) related to P2RX2. (d) Significantly enriched GO terms and KEGG pathways associated with P2RX2.

To better comprehend the biological functions of P2RX2, coexpression analysis was conducted by LinkedOmics, and 6205 genes were positively correlated and 3876 genes were negatively correlated with P2RX2 (Figure 6(a)). The top 50 positively and negatively correlated genes are shown in Figure 6(b) and 6(c). The results of enrichment analysis indicated that P2RX2 coexpressed genes were mainly involved in biological processes, including regulation of chemotaxis and regulation of chemotaxis. KEGG pathway analysis revealed that P2RX2 coexpressed genes mainly participate in aminoacyl-tRNA biosynthesis, homologous recombination, and DNA replication (Figure 6(d)).

### 3.6. Relationship between P2RX2 Expression and Immune Infiltration and TMB

**Figure 7:**
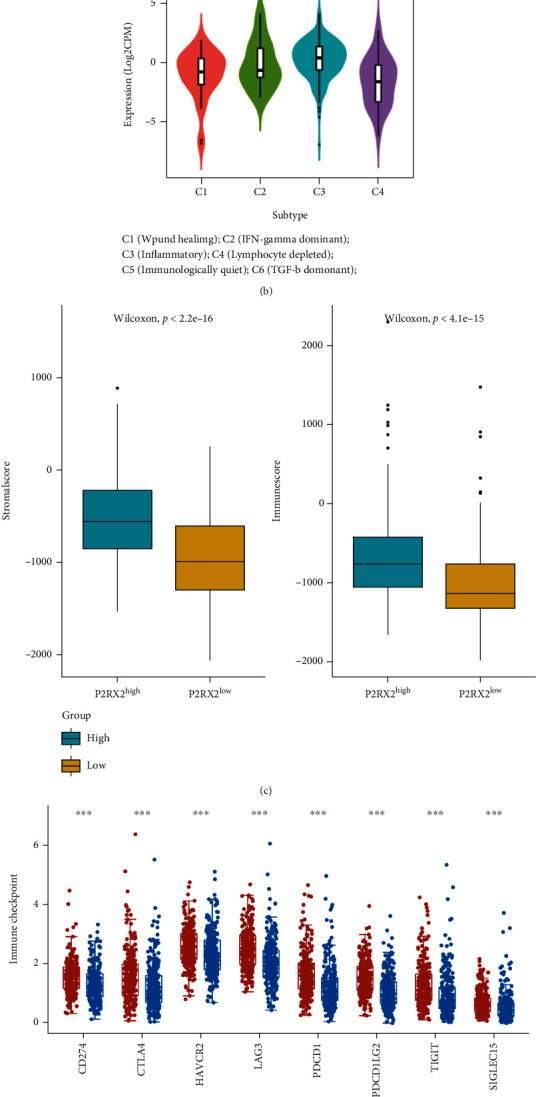
Association between the expression of P2RX2 and immune infiltration in PCa. (a) The proportions of TME cells in the P2RX2^high^ and P2RX2^low^ groups. (b) P2RX2 expression in different immune subtypes. (c) ImmuneScore and StromalScore of the P2RX2^high^ and P2RX2^low^ groups. (d) The expression of immune checkpoint genes between P2RX2^high^ and P2RX2^low^ patients. (e) The association between P2RX2 expression and IPS.

As presented in Figure 7(a), immune cell infiltration was compared between the P2RX2^high^ and P2RX2^low^ groups. A significant difference was observed in the degree of infiltration of all 28 types of immune cells, which was also validated by the results from TIMER (Figure S4). Immune infiltration levels decreased in the P2RX2^low^ group, indicating a poor prognosis. Thereafter, we used the estimation algorithm to calculate the ImmuneScore and StromalScore. Stromal activity was significantly downregulated in the P2RX2^low^ group (Figure 7(c)). According to the results from the TISIDB database, P2RX2 was significantly differentially expressed among the six immune subtypes (Figure 7(b)). Moreover, the expression of immune checkpoint genes, including PD-1, PD-L1, CTLA4, PD-L2, LAG3, TIGIT, and SIGLEC15, was strongly associated with P2RX2 expression (Figure 7(d)). IPS analysis was performed in the two groups to evaluate immunogenicity. The IPS, IPS-CTLA4, IPS-PD1, and IPS-PD1-CTLA4 scores were significantly lower in the P2RX2^low^ group, suggesting a poor response to immunotherapy (Figure 7(e)). Finally, we observed that the TMB was significantly increased in the P2RX2^high^ group compared to that in the P2RX2^low^ group (Figure S5).

### 3.7. Drug Sensitivity Analysis of P2RX2

The correlation between P2RX2 expression and drug sensitivity was examined using the CellMiner database. Among these, 19 drugs were significantly associated with the expression of P2RX2 (Table S2). The top nine genes are presented in Figure S6, and the sensitivity of some antitumor drugs, such as quizartinib, pevonedistat, and apitolisib, was positively correlated with P2RX2 expression, whereas the sensitivity of some drugs, such as rigosertib, was negatively correlated to P2RX2 expression. Moreover, considering the importance of androgen deprivation therapy and chemotherapy in the treatment of PCa, we estimated the IC50 values of Bicalutamide and docetaxel. The results illustrated that the IC50 values of these drugs were significantly lower in the P2RX2^low^ group, suggesting good responsiveness in these patients (Figure S7).

### 3.8. Pan-Cancer Analysis

**Figure 8:**
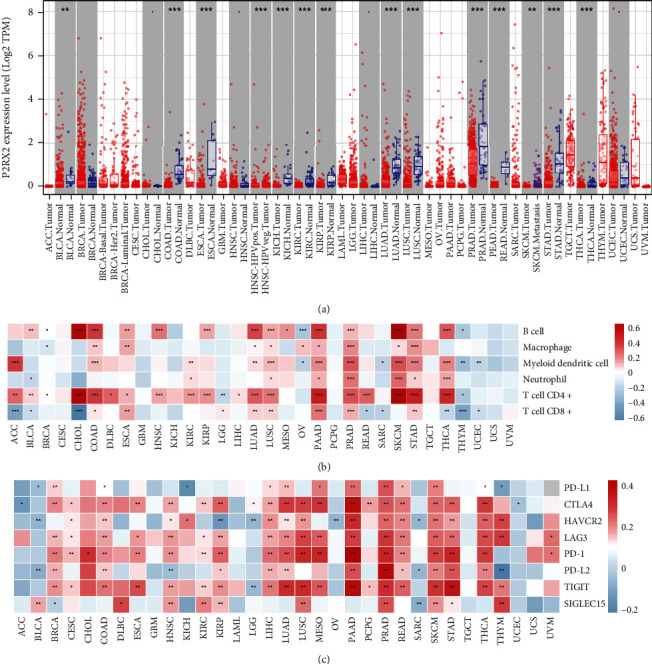
A pan-cancer analysis of P2RX2. (a) The expression of P2RX2 in pan-cancer analyzed by the TIMER dataset. (b) Association between the expression of P2RX2 and the immune infiltration levels in 33 cancer types. (c) Association between the expression of P2RX2 and the expression of immune checkpoint genes in 33 cancer types (^∗^*p* < 0.05, ^∗∗^*p* < 0.01, ^∗∗∗^*p* < 0.001).

The mRNA expression of P2RX2 in human cancers was analyzed using the TIMER online database. Differential expression of P2RX2 was observed in BLCA, COAD, ESCA, HNSC, KICH, KIRC, KIRP, LUAD, LUSC, READ, STAD, THCA, and PRAD (Figure 8(a)). Finally, the correlation of P2RX2 with immune cells and checkpoint genes is presented in Figures 8(b) and 8(c). P2RX2 is strongly associated with different immune cells and checkpoints.

### 3.9. GSEA Analysis of P2RX2

GSEA was conducted between the P2RX2^high^ and P2RX2^low^ groups. The results indicated that the downregulation of P2RX2 may activate pathways promoting cancer progression, such as the PI3K − Akt, NF − kappa B, and JAK − STAT signaling pathways (Figure S8).

### 3.10. Correlation of P2RX2 Expression with PCa Cell Malignant Features

**Figure 9:**
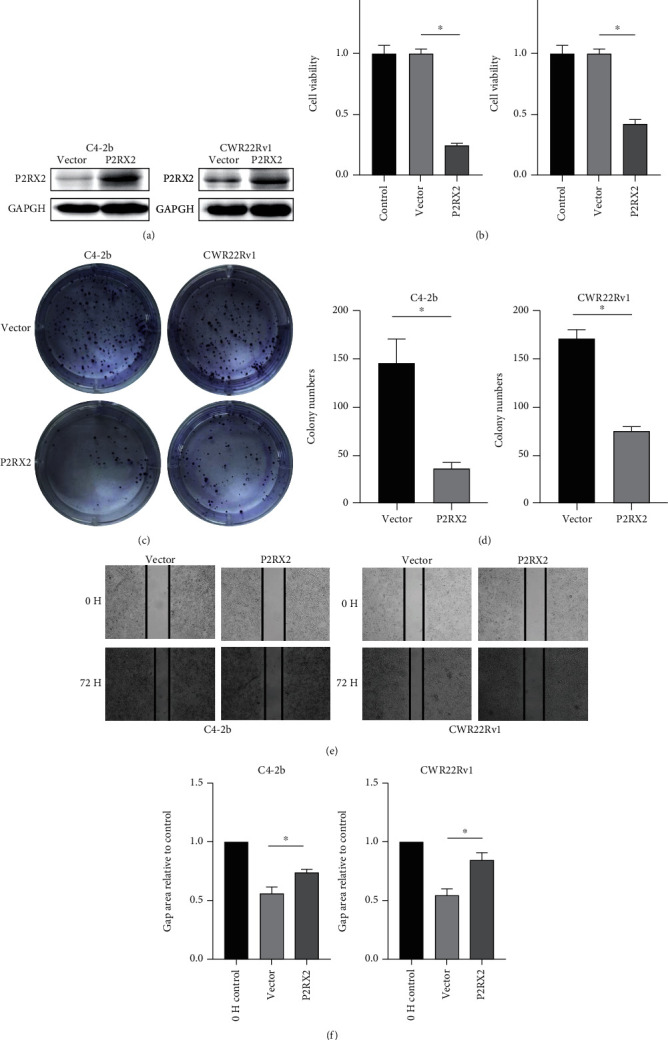
P2RX2 acts as a tumor suppressor in PCa: (a) The expression of P2RX2 in C4-2b and CWR22Rv1 cells after transfection. (b) Cell counting kit-8 for C4-2b and CWR22Rv1 cells after 24 h. (c–d) Scratch assay in the C4-2b and CWR22Rv1 cells. (f–g) Colony formation in C4-2b and CWR22Rv1 cells.

To validate the role of P2RX2 as a tumor suppressor gene in PCa, we overexpressed P2RX2 in PCa cell lines by transfecting plasmids and verified the upregulation of P2RX2 using western blotting (Figure 9(a)). Subsequently, the CCK-8 results revealed that the upregulation of P2RX2 significantly suppressed the proliferation of PCa cells (Figure 9(b)). Moreover, the colony formation assay indicated that P2RX2 overexpression remarkably reduced the viability of PCa cells compared to that in the control groups (Figures 9(c) and 9(d)). To determine the effect of P2RX2 upregulation on PCa cell migration, a scratch assay was performed. Similar to previous outcomes, upregulation of P2RX2 decreased the migration of PCa cells (Figures 9(e) and 9(f)).

## 4. Discussion

Calcium channels participate in many cellular processes such as proliferation, differentiation, and apoptosis [34]. Among the mechanisms dysregulated in cancer, those associated with calcium ions (Ca2+) play critical roles in various dimensions of tumors [35]. Some aggressive features of tumors, such as metastatic dissemination, correlate with alterations in calcium homeostasis in cancer cells [36]. Treatment with calcium channel blockers has been reported to decrease tumor cell growth, and the combination of calcium channel blockers and antiestrogens can reverse resistance to antiestrogens in breast cancer [37]. It is widely acknowledged that calcium channels have an impact on tumor progression in PCa. Calcium channel blockers have been demonstrated to influence tumor progression [34]. Some hub genes related to calcium channels reportedly play significant roles in several cancers and have been associated with patient prognosis [38–40]. Therefore, performing an integrated analysis of calcium channel-related genes is necessary.

In this study, we obtained calcium channel-related genes from the GO website and identified the differentially expressed genes between PCa and normal tissues. Nine genes were obtained after prognostic analysis, and P2RX2, which was closely correlated with immune infiltrating cells and immune checkpoint gene expression, was selected for further analysis. The prognostic value and differential expression of P2RX2 in PCa and normal tissue samples were validated using GEPIA. Clinical correlation analysis demonstrated that P2RX2 was negatively correlated with TNM stages, Gleason score, and PFI events. Additionally, we also analyzed the gene mutations of P2RX2 in PCa, and missense and synonymous substitutions were the main genetic alterations. After performing a coexpression analysis of P2RX2, the genes coexpressed with P2RX2 were obtained and used for the GO and KEGG enrichment analyses. These results indicated that P2RX2 might be involved in pathways such as DNA replication, cell cycle, and cytokine–cytokine receptor interaction.

Many studies have revealed that infiltrating immune cells in the tumor microenvironment affect the development and progression of tumors [41, 42]. Noncancerous cells in the tumor microenvironment, such as immune cells and fibroblasts, may influence the response of cancer cells to treatment [43]. Calcium signaling plays a critical role in various cellular functions of the immune system. For instance, the engagement of T-cell and B-cell antigen receptors requires an increase in intracellular Ca2+ concentration during an immune response [15]. Here, we assessed the correlation between immune infiltration cells and P2RX2 expression and observed that the infiltration levels of all 28 immune cells decreased in patients with low expression of P2RX2. Similarly, the ImmuneScore and StromalScore were lower in the P2RX2^low^ group. Thus, the escape of tumor cells from the immune system may lead to poor survival in these patients. In recent years, immunotherapy has revolutionized cancer therapeutic regimens and has become a vital strategy for treating patients with advanced cancers [44]. Several studies have suggested that the combination of immune checkpoint blockade (ICB) with traditional therapeutics, such as androgen deprivation therapy, radiotherapy, and chemotherapy, can enhance immune responses and induce long-lasting clinical responses [45]. Therefore, we evaluated the correlation between immune checkpoint gene expression and P2RX2 expression. P2RX2 expression was significantly positively correlated with the expression of all immune checkpoint genes evaluated, including PD-1, CTLA4, PD-L1, and PD-L2. Additionally, we predicted the potential ICB response in patients using IPS analysis, and the IPS score of the P2RX2^low^ group was significantly lower than that of the P2RX2^high^ group. Hence, P2RX2 may play an instrumental role in tumor immunity and act as a therapeutic target to enhance patient response to immunotherapy in PCa.

Androgen deprivation therapy and chemotherapy (docetaxel) remain the first-line treatment options for PCa [46]. We used the GDSC database to predict the IC50 values of the drugs used for the treatment of PCa. We observed that patients with low P2RX2 expression had a higher predicted IC50 value for both docetaxel and Bicalutamide, suggesting a higher sensitivity to these treatments. Finally, we conducted a pan-cancer analysis, and the results indicated that P2RX2 is differentially expressed in several cancers, such as BLCA, COAD, KIRC, LUAD, and PAAD. Moreover, we correlated P2RX2 expression with immune cell infiltration and expression of immune checkpoint genes. The results revealed that P2RX2 was closely related to immune cell infiltration in various tumors, especially LUAD, LUSC, PAAD, PRAD, SKCM, STAD, and THCA. Moreover, P2RX2 may play a vital role in oncogenesis and altering the immune microenvironment. Additionally, in-vitro molecular experiments were performed. The results revealed that upregulation of P2RX2 significantly decreased PCa cell migration, proliferation, and colony formation, suggesting a key role of P2RX2 in PCa.

Although we identified a hub gene, P2RX2, from the comprehensive analysis of calcium channel-related genes, several limitations also exist. First, our outcomes were obtained from an analysis of public databases, and the prognostic value of P2RX2 needs to be validated in prospective cohorts. Second, the effect of P2RX2 on tumor immune infiltration should be further validated by molecular and animal experiments. Finally, as the data currently available in the GDSC database are limited, we could not estimate the IC50 values of abiraterone and enzalutamide in our study, which are the first-line therapies for metastatic castration-resistant PCa [46–48].

## 5. Conclusion

We performed an integrated analysis of calcium channel-related genes and identified the hub gene P2RX2, which might be a novel prognostic biomarker in PCa. P2RX2 has a significant positive correlation with immune cell infiltration and the expression of immune checkpoint genes in PCa. A lower IPS score in patients with low P2RX2 expression indicated a poor response to immunotherapy in these patients. However, patients with low P2RX2 expression may be more sensitive to docetaxel and Bicalutamide.

## Figures and Tables

**Table 1:** Correlations between P2RX2 expression and clinicopathological parameters.

Characteristic	Low expression of P2RX2	High expression of P2RX2	p
*n*	249	250	
T stage, *n* (%)			0.010
T2	78 (15.9%)	111 (22.6%)	
T3	160 (32.5%)	132 (26.8%)	
T4	7 (1.4%)	4 (0.8%)	
N stage, n (%)			0.125
N0	175 (41.1%)	172 (40.4%)	
N1	48 (11.3%)	31 (7.3%)	
M stage, *n* (%)			0.248
M0	228 (49.8%)	227 (49.6%)	
M1	3 (0.7%)	0 (0%)	
Primary therapy outcome, *n* (%)			0.966
PD	15 (3.4%)	13 (3%)	
SD	14 (3.2%)	15 (3.4%)	
PR	19 (4.3%)	21 (4.8%)	
CR	168 (38.4%)	173 (39.5%)	
Age, *n* (%)			0.682
< = 60	109 (21.8%)	115 (23%)	
>60	140 (28.1%)	135 (27.1%)	
PSA (ng/ml), *n* (%)			0.456
<4	207 (46.8%)	208 (47.1%)	
> =4	16 (3.6%)	11 (2.5%)	
Gleason score, *n* (%)			< 0.001
6	15 (3%)	31 (6.2%)	
7	107 (21.4%)	140 (28.1%)	
8	37 (7.4%)	27 (5.4%)	
9	88 (17.6%)	50 (10%)	
10	2 (0.4%)	2 (0.4%)	
OS event, *n* (%)			0.063
Alive	241 (48.3%)	248 (49.7%)	
Dead	8 (1.6%)	2 (0.4%)	
PFI event, *n* (%)			0.049
Alive	193 (38.7%)	212 (42.5%)	
Dead	56 (11.2%)	38 (7.6%)	

## Data Availability

The data and materials can be obtained by contacting the corresponding author.

## Supplementary Materials

Supplementary Materials Figure S1: volcano plot of the 100 significantly differentially expressed genes. Figure S2: the P2RX2 expression between different BCR statuses. Figure S3: P2RX2 expression in adjacent normal tissue and prostate cancer. Figure S4: correlation analysis of P2RX2 expression and abundance of immune cells in TIMER. Figure S5: the difference in TMB between the P2RX2^high^ and P2RX2^low^ groups. Figure S6: drug sensitivity analysis of P2RX2 by CellMiner. Figure S7: the IC50 of Docetaxel and Bicalutamide between P2RX2^high^ and P2RX2^low^ groups. Figure S8: GSEA analysis between P2RX2^high^ and P2RX2^low^ groups.
