# Supplementary material for: Identification of Calcium Channel-Related Gene P2RX2 for Prognosis and Immune Infiltration in Prostate Cancer
Source: Dis Markers. 2022 Sep 30;2022:8058160. doi: 10.1155/2022/8058160 (PMC9553555; doi:10.1155/2022/8058160)
Supplement: Supplementary Materials — Figure S1: volcano plot of the 100 significantly differentially expressed genes. Figure S2: the P2RX2 expression between different BCR statuses. Figure S3: P2RX2 expression in adjacent normal tissue and prostate cancer. Figure S4: correlation analysis of P2RX2 expression and abundance of immune cells in TIMER. Figure S5: the difference in TMB between the P2RX2high and P2RX2low groups. Figure S6: drug sensitivity analysis of P2RX2 by CellMiner. Figure S7: the IC50 of Docetaxel and Bicalutamide between P2RX2high and P2RX2low groups. Figure S8: GSEA analysis between P2RX2high and P2RX2low groups. [file 8058160.f1.zip › supplementary figures (2).pdf]

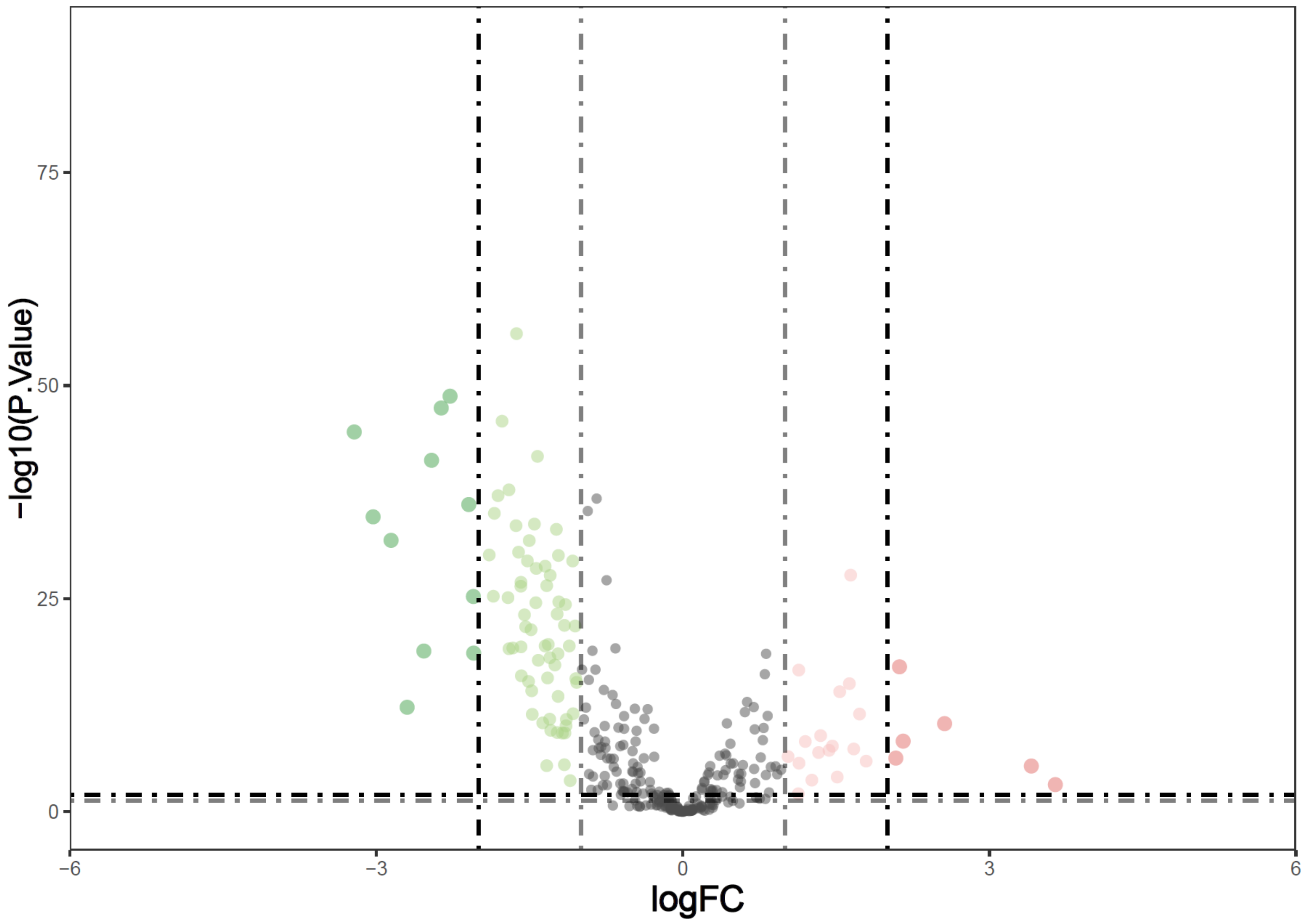

Figure S1: Volcano plot of the 100 significantly differentially expressed genes

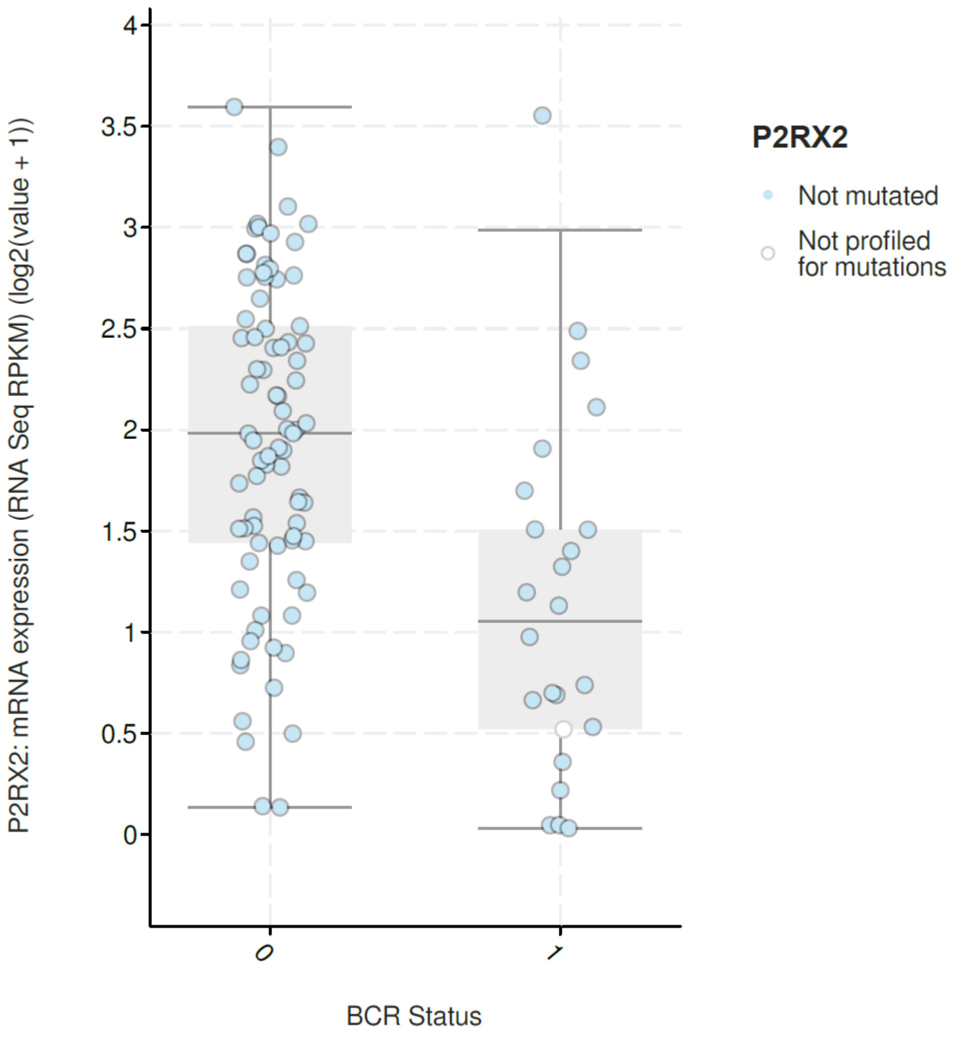

Figure S2: The P2RX2 expression between different BCR status

normal

Pca (Gleason score =6)

Pca (Gleason score =9)

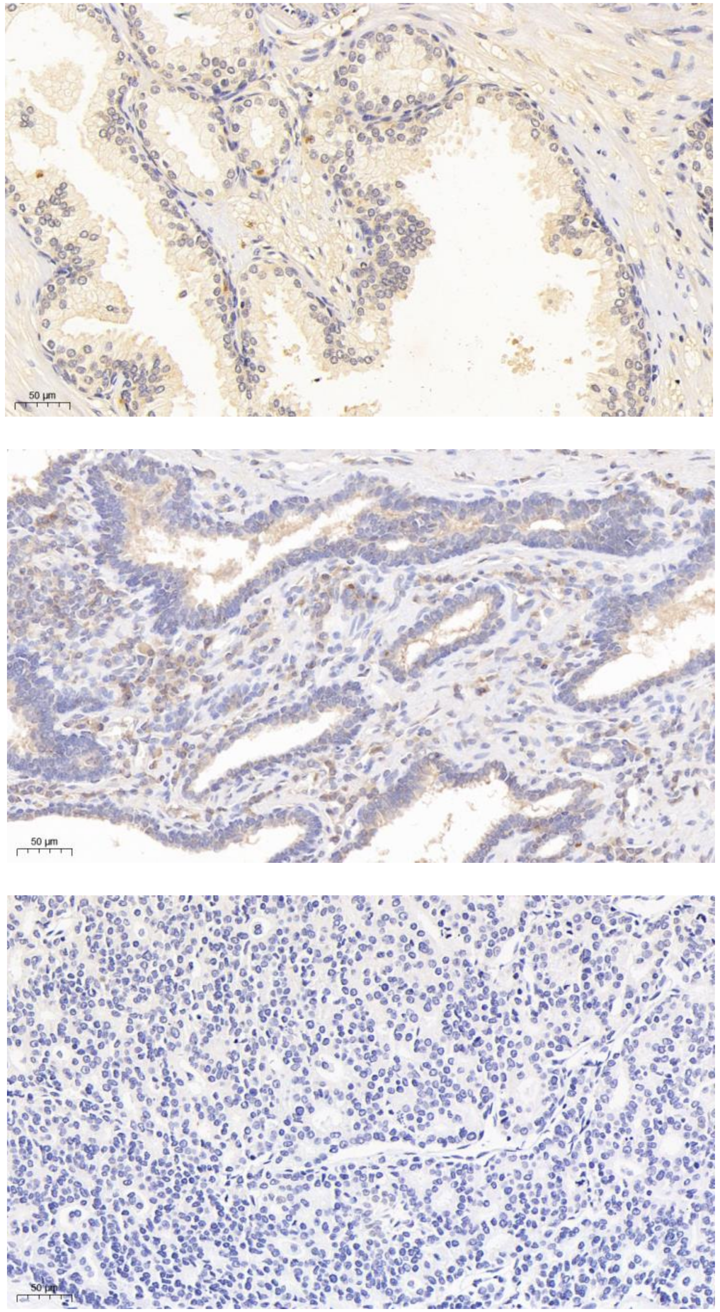

Figure S3: P2RX2 expression in adjacent normal tissue and prostate cancer

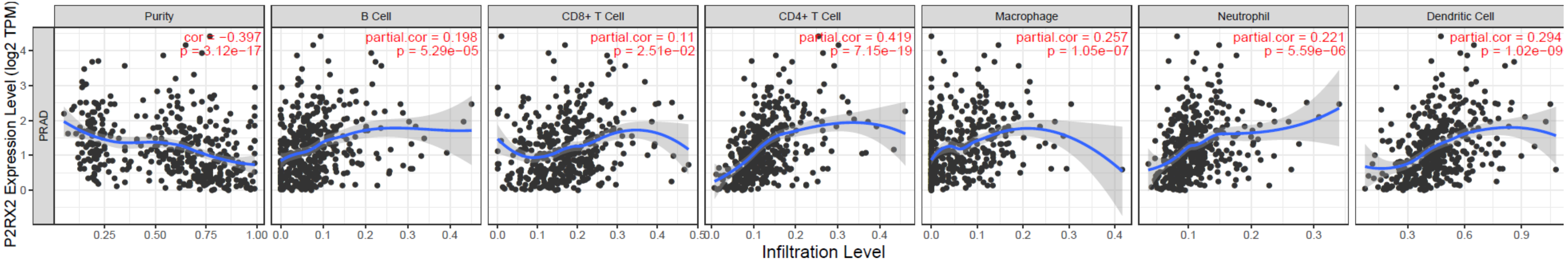

Figure S4: Correlation analysis of P2RX2 expression and abundance of immune cells in TIMER.

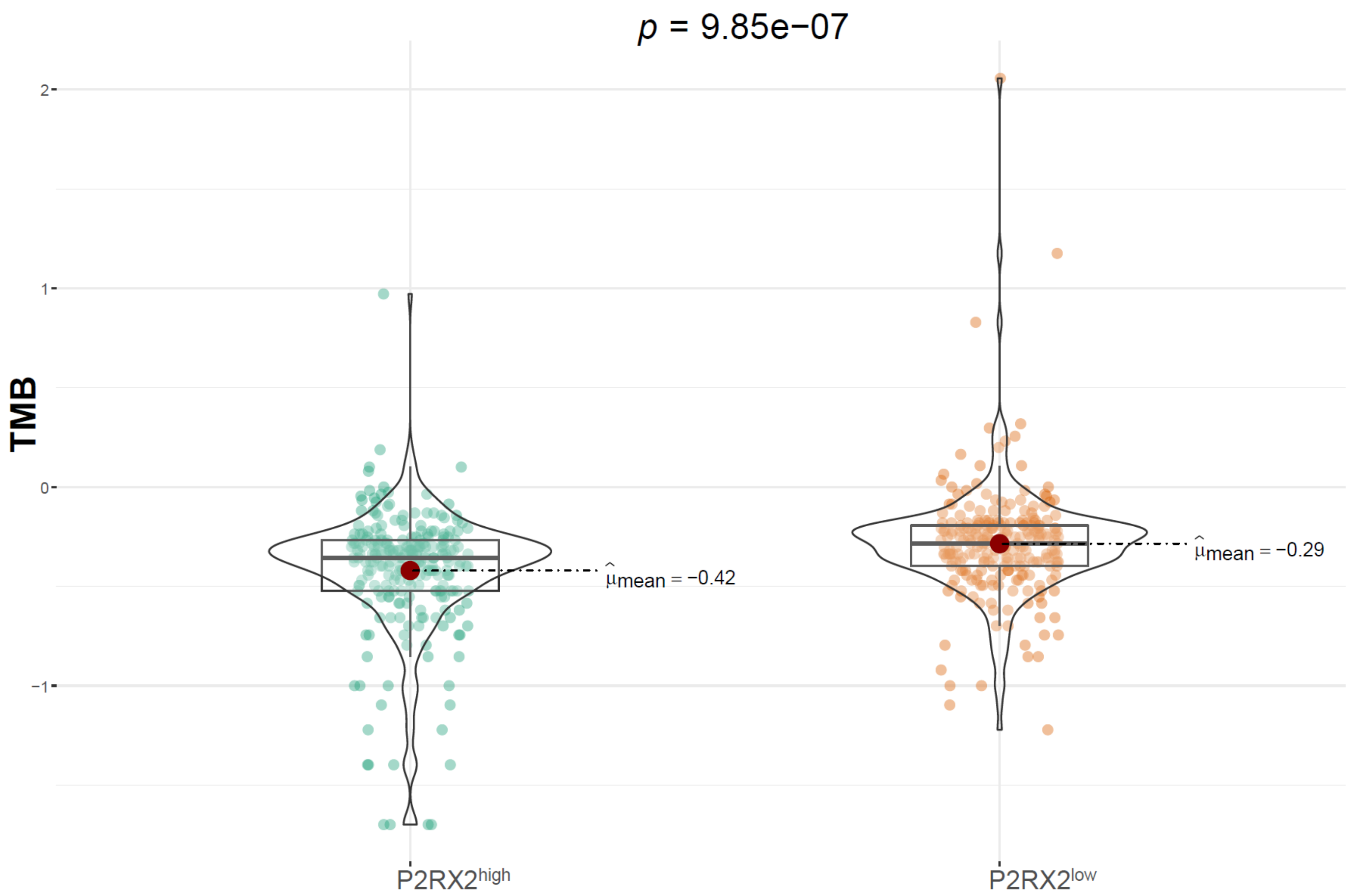

Figure S5: The difference in TMB between the P2RX2^{high} and P2RX2^{low} groups

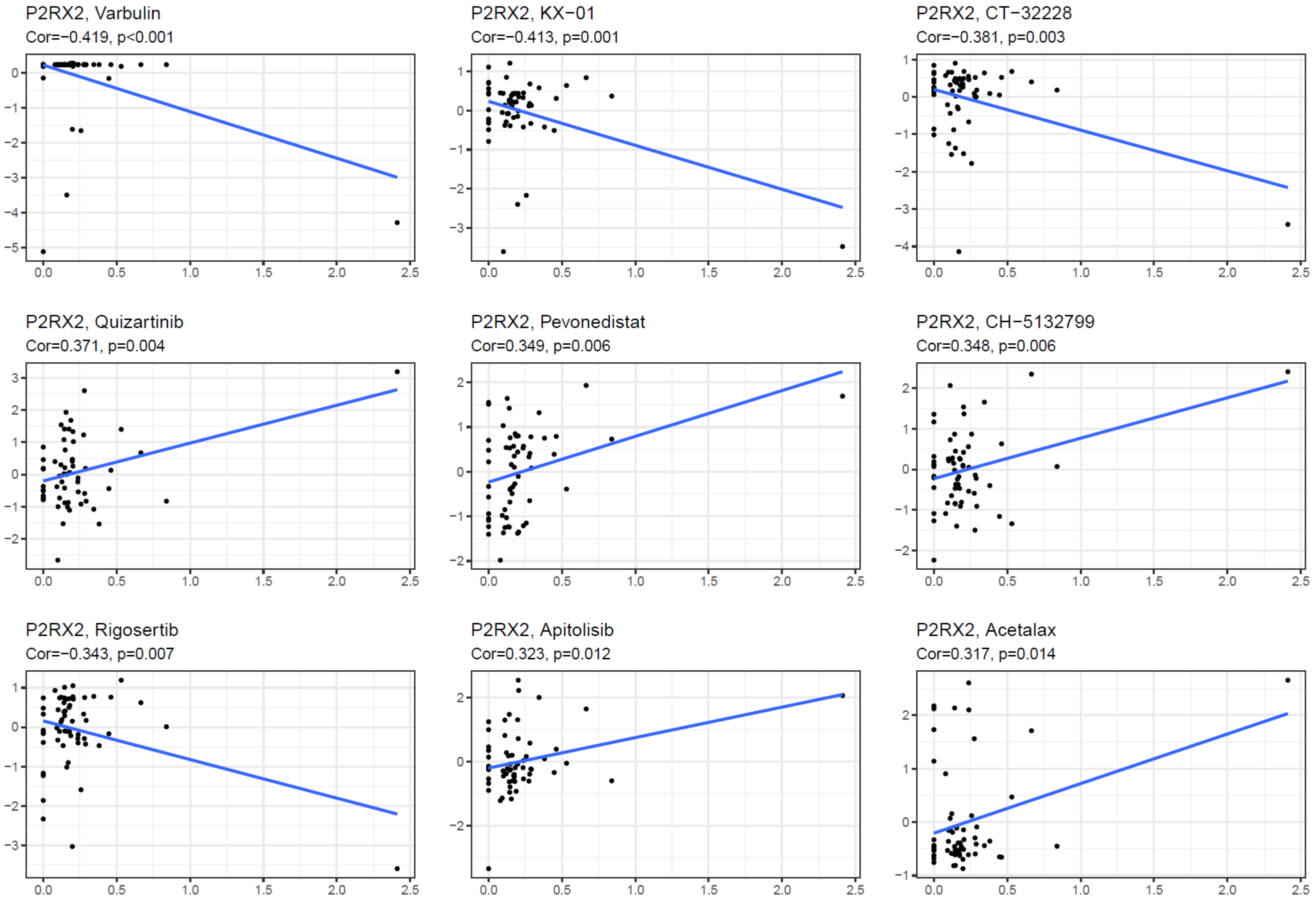

Figure S6: drug sensitivity analysis of P2RX2 by CellMiner

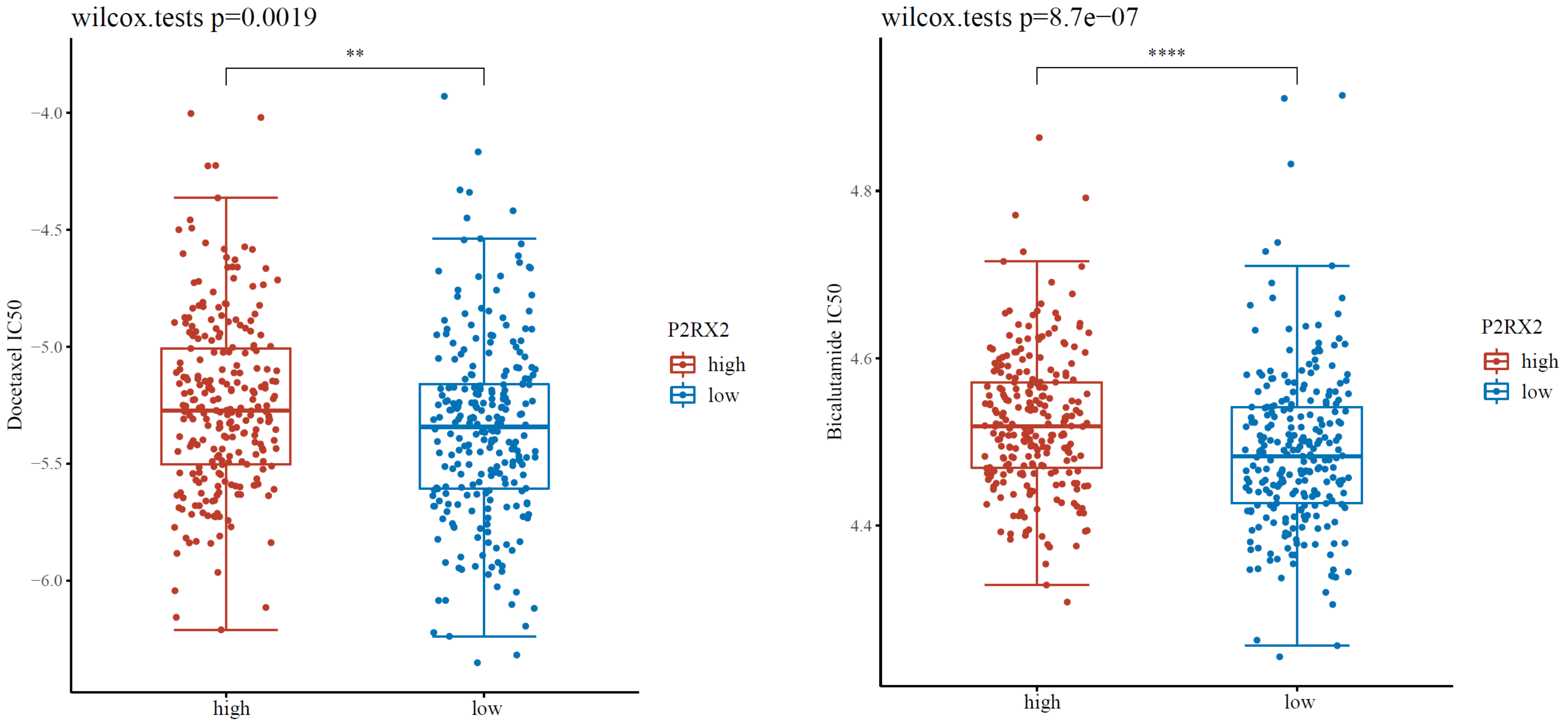

Figure S7: The IC50 of Docetaxel and Bicalutamide between P2RX2^{high} and P2RX2^{low} groups.

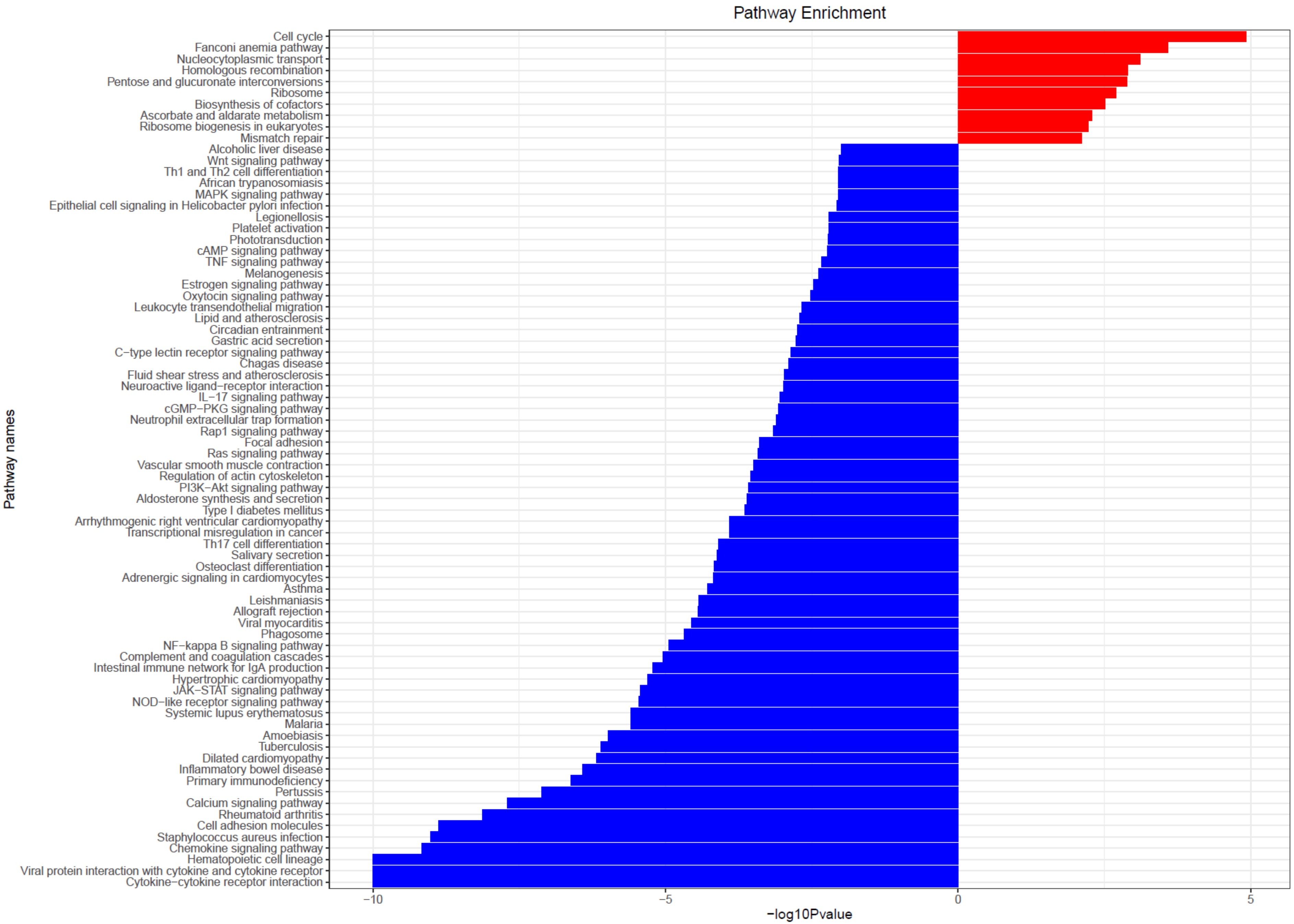

Figure S8: GSEA analysis between P2RX2^{high} and P2RX2^{low} groups.
