# Supplementary material for: Identification of Calcium Channel-Related Gene P2RX2 for Prognosis and Immune Infiltration in Prostate Cancer
Source: Dis Markers. 2022 Sep 30;2022:8058160. doi: 10.1155/2022/8058160 (PMC9553555; doi:10.1155/2022/8058160)
Supplement: Supplementary Materials — Figure S1: volcano plot of the 100 significantly differentially expressed genes. Figure S2: the P2RX2 expression between different BCR statuses. Figure S3: P2RX2 expression in adjacent normal tissue and prostate cancer. Figure S4: correlation analysis of P2RX2 expression and abundance of immune cells in TIMER. Figure S5: the difference in TMB between the P2RX2high and P2RX2low groups. Figure S6: drug sensitivity analysis of P2RX2 by CellMiner. Figure S7: the IC50 of Docetaxel and Bicalutamide between P2RX2high and P2RX2low groups. Figure S8: GSEA analysis between P2RX2high and P2RX2low groups. [file 8058160.f1.zip › Table S2 (1).docx]

Table S2: Drugs significantly associated with the expression of P2RX2

| Gene | Drug | cor | pvalue |
| --- | --- | --- | --- |
| P2RX2 | Varbulin | -0.41948 | 0.000849 |
| P2RX2 | KX-01 | -0.41346 | 0.001025 |
| P2RX2 | CT-32228 | -0.38114 | 0.00266 |
| P2RX2 | Quizartinib | 0.370761 | 0.003543 |
| P2RX2 | Pevonedistat | 0.349379 | 0.006216 |
| P2RX2 | CH-5132799 | 0.348343 | 0.006382 |
| P2RX2 | Rigosertib | -0.34283 | 0.00733 |
| P2RX2 | Apitolisib | 0.322744 | 0.011901 |
| P2RX2 | Acetalax | 0.317094 | 0.013564 |
| P2RX2 | pralatrexate | -0.31131 | 0.01547 |
| P2RX2 | TRICIRIBINE PHOSPHATE | 0.309466 | 0.016125 |
| P2RX2 | Eribulin mesilate | -0.30362 | 0.018357 |
| P2RX2 | MK-2206 | 0.297934 | 0.020777 |
| P2RX2 | Elesclomol | 0.286256 | 0.026602 |
| P2RX2 | Sonidegib | 0.28327 | 0.028294 |
| P2RX2 | 6-(4-pyrimidinyl)-1H-indazole derivative | 0.281173 | 0.029536 |
| P2RX2 | Copanlisib | 0.261551 | 0.043525 |
| P2RX2 | Etoposide | 0.257973 | 0.046587 |
| P2RX2 | PF-477736 | -0.25634 | 0.048037 |
